# Predictive Distribution Modeling of the Medicinal Leech *Hirudo verbana* Carena, 1820 (Hirudinea, Hirudinidae) in Sicily: Implications for Conservation

**DOI:** 10.1002/ece3.72410

**Published:** 2025-11-02

**Authors:** Mirko Liuzzo, Serge Utevsky, Federico Marrone

**Affiliations:** ^1^ Department of Environmental Sciences, Informatics and Statistics (DAIS), Ca' Foscari University of Venice Venice Italy; ^2^ Université de Strasbourg, CNRS, IPHC UMR7178 Strasbourg France; ^3^ Department of Zoology and Animal Ecology, School of Biology V.N. Karazin Kharkiv National University Kharkiv Ukraine; ^4^ Department of Biological, Chemical and Pharmaceutical Sciences and Technologies (STEBICEF), University of Palermo Palermo Italy

**Keywords:** BIOMOD2, habitat suitability, near‐threatened species, protected areas, wetland

## Abstract

*Hirudo verbana*, a medicinal leech species of conservation concern, has long been considered rare and fragmented across its distribution range due to overexploitation and habitat alteration. In this study, we present the first predictive distribution model for *H. verbana* in Sicily, combining field occurrence data (time: 1980–2024) with environmental variables using an ensemble species distribution modeling (SDM) framework. Unlike its typical occurrence in temperate lowland wetlands of central‐eastern Europe, the species in Sicily shows a marked shift toward upper hill and montane zones. Species presence was strongly associated with high NDVI values, reflecting a preference for areas with dense and structurally complex vegetation. In contrast, presence probability declined sharply with increasing terrestrial human footprint (THF), indicating a notable sensitivity to anthropogenic disturbance. Aquatic habitat type also emerged as a key predictor: the species was most likely to occur in lentic environments such as standing water bodies, and least likely in lotic systems or areas lacking aquatic habitats. Notably, most high‐suitability areas overlapped with sites in the Natura 2000 network, emphasizing the importance of existing protected areas as refugia for the species. Moreover, some previously undocumented areas of high habitat suitability were identified, providing a spatially basis for refining monitoring strategies and informing conservation planning for this ecologically sensitive freshwater invertebrate.

## Graphical abstract

A predictive distribution model for *Hirudo verbana* in Sicily was developed using 1980–2024 occurrence data and environmental variables. The species is associated with densely vegetated, high‐elevation lentic habitats and avoids human‐disturbed areas. Natura 2000 sites and newly identified suitable zones emerge as key areas for targeted conservation.

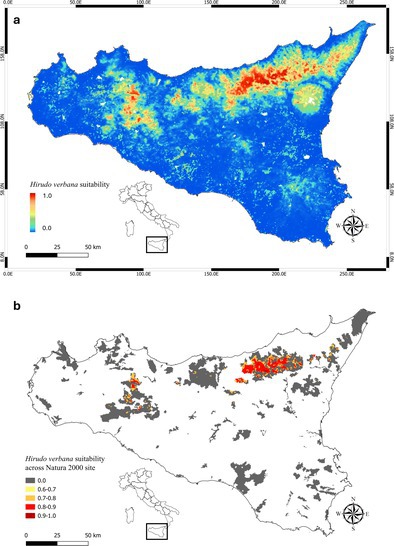

## 1 Introduction

Wetlands are among the most productive and ecologically significant ecosystems on the planet, serving as key ecotones where terrestrial and aquatic processes interact (Mitsch et al. 2015). They provide a wide array of ecological functions, including water filtration, flood mitigation, carbon sequestration, and shoreline stabilization (Xu et al. 2020). Moreover, wetlands provide essential habitats for a variety of flora and fauna, many of which are highly specialized and depend exclusively on these environments for their survival (Baigún et al. 2022). As a result, wetlands are unanimously recognized as biodiversity hotspots, supporting disproportionately high levels of species richness compared to their global extent (Dudgeon et al. 2006; Viaroli et al. 2016). Despite their importance, wetlands are among the most threatened ecosystems worldwide, with significant losses due to human activities such as agriculture, urban development, biological invasions, and pollution (Liuzzo et al. 2018; Leberger et al. 2020; Haase et al. 2023). Alterations in hydrological regimes, water pollution, overexploitation, biological invasions and the widespread loss of natural habitats represent some of the most significant threats to wetland ecosystems, to which some emergent threats should also be added (Reid et al. 2019). Approximately 60% of species associated with these environments have undergone population declines as a direct result of anthropogenic pressures (Ramsar Convention Secretariat 2018). This alarming trend highlights the critical need for robust and targeted conservation strategies aimed at mitigating such impacts and ensuring the long‐term ecological integrity of the best‐preserved wetlands.

In this context, medicinal leeches of the genus *Hirudo* are threatened across their distribution range as a result of overexploitation and habitat alteration. Medicinal leeches were in fact central to classical phlebotomy and the widespread practice of therapeutic blood‐letting in Europe, and their salivary bioactive compounds remain of scientific interest today (Elliott and Kutschera 2011). Recent taxonomic revisions have revealed that what was once regarded as a single species actually comprises several distinct taxa, including *H. verbana*. A critical reassessment of historical records concerning medicinal leeches is thus needed since these did not account for the true diversity within the group and the possible role of human‐mediated translocations in shaping the currently observed diversity pattern (Sağlam et al. 2016; Arias et al. 2021).

The diversity of the genus is high and to date not fully understood, and in the last decades, several different taxa previously lumped under *Hirudo medicinalis* were resurrected or described ex novo (e.g., Utevsky and Trontelj 2005; Sağlam et al. 2016; DarabiDarestani et al. 2018; Wang et al. 2022), so that an accurate knowledge of the species distribution is to date missing. Based on currently available data, at least five native *Hirudo* species occur in the West‐Palaearctic area: *Hirudo medicinalis* in central, western, and northern Europe, *H. orientalis* occurring from the Caucasus to Kazakhstan, Uzbekistan, and Iran, *H. sulukii* in Turkey, *H. troctina* in the Maghreb, Sardinia, and Iberian Peninsula, and *H. verbana* in southern Europe, Asia Minor, and Central Asia. This last species is characterized by a noteworthy molecular and phenotypical structuring, with at least three distinct lineages occurring parapatrically (Utevsky and Trontelj 2016; Arias et al. 2021). The autoecology of the different *Hirudo* species and lineages is to date insufficiently known, although there is some evidence suggesting that they have different ecological niches and feeding habits (e.g., Laufer et al. 2008, Kovalenko and Utevsky 2012, Vecchioni et al. 2025, and references therein).

In the light of their poor conservation status, all the *Hirudo* species occurring in Europe are included *sub Hirudo medicinalis* in Annex V of the “Habitats Directive” of the European Union (Council Directive 92/43/EEC of 21 May 1992), in Appendix III of the Bern Convention on the Conservation of European Wildlife and Natural Habitats, and in Appendix II of CITES (Convention on International Trade in Endangered Species of Wild Fauna and Flora).

In Italy, the occurrence of at least two *Hirudo* species is known; these are *Hirudo troctina*, reported for Sardinia (Marrone et al. 2024), and *Hirudo verbana*, occurring in mainland Italy and Sicily (Marrone et al. 2021). Populations from Apulia have been assigned to the “western clade” of *H. verbana* based on molecular data (Trontelj and Utevsky 2005; Vecchioni et al. 2025). However, genetic information remains limited for most other mainland Italian and Sicilian populations traditionally attributed to *H. verbana*. The pronounced inter‐populational color polymorphism in Italian *Hirudo* populations further suggests that additional, as yet uncharacterized, lineages may occur in these areas (F. Marrone unpublished data). Nevertheless, mitochondrial and nuclear DNA markers consistently recover well‐supported monophyletic clades within the genus *Hirudo*, and all the available genetic sequences from southern Italy cluster within the *H. verbana* clade (Trontelj and Utevsky 2005; Siddall et al. 2007; Elliott and Kutschera 2011; Vecchioni et al. 2025, F. Marrone unpublished data). Regardless of their species‐level identity, all populations of the genus *Hirudo* present in Italy are considered to be experiencing “a significantly (or rather, dramatically) negative trend” throughout the country (Minelli 2006).

Understanding the ecological requirements of *Hirudo* species is key to species conservation efforts, particularly due to the paucity of comprehensive distribution records, which prevents the realization of large‐scale informed management of the species (Utevsky et al. 2010; Marrone and Canale 2019; Marrone et al. 2021). Species Distribution Models (SDMs) serve as a powerful approach to evaluate relationships between species occurrence and environmental predictors that regulate it (Goldenberg et al. 2024), thereby enabling the estimation of a species' potential geographic range (Elith et al. 2011; Renner and Warton 2013). These models are extensively used in conservation science to identify suitable habitats, assess habitat quality, and guide the development of management strategies for at‐risk species across different spatial scales (Williams et al. 2009; Hunt et al. 2020). In the case of *H. verbana* populations in Sicily, SDMs might allow for a characterization of their potential distribution supporting the planning of targeted surveys and methodologically sound census efforts.

Given the urgent need for effective conservation measures for *H. verbana*, this study aims to: (i) analyze its distribution in Sicily based on available occurrence records; (ii) characterize its ecological requirements by evaluating key environmental variables, including topographical, ecological, and climatic factors; (iii) assess the extent and spatial distribution of suitable habitats within the Natura 2000 network in Sicily using SDMs; and (iv) discuss the conservation implications for *H. verbana* within the context of the progressive degradation of inland water ecosystems in the Mediterranean region.

## 2 Materials and Methods

### 2.1 Study Area

Sicily, located at the centre of the Mediterranean Basin, exhibits a wide range of bioclimatic conditions due to its complex topography, spanning from mountainous interiors to extensive coastal plains. This variability supports a wide array of wetland ecosystems, including brackish coastal ponds, lagoons, rivers, and both natural and artificial inland wetlands, which serve as crucial habitats for numerous resident and migratory species (Ferrarini et al. 2021; Marrone and NaselliFlores 2022). The regional human population is primarily concentrated in coastal areas and major urban centres such as Palermo and Catania, resulting in a population density of approximately 195 inhabitants per km^2^ (ISTAT 2023). This demographic pattern places considerable anthropogenic pressure on nearby wetland environments (Ferrarini et al. 2022).

Sicily is also home to 238 Natura 2000 sites encompassing both terrestrial and marine areas. The terrestrial sites cover 469,847 ha (~4698 km^2^), while the marine sites extend over 169,288 ha (~1693 km^2^) (ARPA Sicilia 2020). The island's Mediterranean climate is characterized by considerable spatial variability, with mean annual temperatures ranging from 14°C to 21°C and annual precipitation levels between 400 mm in the arid southern lowlands and over 1200 mm in the northeastern highlands (Sistema Nazionale per la Protezione dell'Ambiente 2023). These climatic gradients present both challenges and opportunities for the effective conservation and management of Sicilian wetland ecosystems.

Sicilian leech fauna has been to date poorly investigated (e.g., Minelli 2006, and references therein), and only a few iconic species of the families Hirudinidae and Glossiphoniidae, like *Hirudo verbana* and *Placobdella costata*, have been the object of recent studies (e.g., Marrone et al. 2016; Marrone and Canale 2019; Vecchioni et al. 2021; Kvist et al. 2022, and references therein).

### 2.2 Distribution Data

Due to the possible presence of multiple *Hirudo* species and evolutionary lineages with distinct ecological traits across different regions of Italy, this study specifically focuses on the populations from Sicily, a biogeographically rather homogeneous area where we expect to find a single taxonomic entity. Distribution data of *H. verbana* were compiled from literature sources (Utevsky et al. 2010; Sorgi et al. 2011; Marrone and Canale 2019; Marrone et al. 2021), unpublished records, personal communications from freshwater fauna specialists, and the social network iNaturalist (Figure S1 and Table S1). To reduce common issues associated with taxonomic uncertainty and spatial inaccuracies, presence records were carefully verified in the field and selected according to established recommendations (Guisan et al. 2007; Tessarolo et al. 2014; Araújo et al. 2019). This process included the elimination of duplicate entries and the correction or exclusion of erroneous records to minimize sampling bias. Such bias, which poses a significant concern in species distribution modeling (Phillips et al. 2006), can introduce spatial autocorrelation and artificially cluster observations, violating assumptions of data independence (Dormann et al. 2007). This process ensured that the occurrence data adequately represented the species' known distribution while maintaining spatial precision consistent with the resolution of the environmental predictor layers. All georeferenced records were incorporated into a Geographic Information System (QGIS) for spatial and graphic analysis (Table S1). The final dataset was used to estimate the habitat suitability and species distribution across Sicily.

### 2.3 Environmental Predictors

Ten bioclimatic variables were initially considered as potential predictors of *Hirudo verbana* habitat distribution, based on previous studies of leech habitat suitability and the predictive performance of species distribution models (Utevsky et al. 2010, Barbet‐Massin et al. 2012, Glombová and Schenková 2015, Popa et al. 2024).

Given that the Mediterranean medicinal leech (*H. verbana*) inhabits diverse freshwater environments and occurs in habitats that offer suitable vertebrate hosts (Marrone et al. 2021; Vecchioni et al. 2025), three categories of variables were considered (Table 1): (1) Climatic variables (downloaded from the WorldClim Database version 2.1, www.worldclim.org/data/worldclim21.html); (2) Topographical variables (altitude, downloaded from the Istituto Nazionale di Geofisica e Vulcanologia (INGV), https://tinitaly.pi.ingv.it/#), and (3) Ecological variables normalized difference vegetation index (NDVI), terrestrial human footprint (THF), and waterways and water bodies occurrence (WWB) obtained from various sources (see Table 1). Multicollinearity among the set of 10 bioclimatic variables was addressed, as it is known to distort the estimation of model parameters and the assessment of variable importance (Dormann 2007; Crase et al. 2012). A correlation matrix was constructed, and for pairs of variables exhibiting a Pearson correlation coefficient |*r*| > 0.7 (Zhang et al. 2024), the variable considered more relevant to the species' life‐history traits was retained (Utevsky et al. 2010; Popa et al. 2024; Dormann et al. 2013; Brandt et al. 2017). All environmental layers were reprojected to the ETRS89/ETRS‐LAEA coordinate reference system and resampled to a 500 m^2^ resolution using bilinear interpolation for continuous variables and nearest‐neighbor interpolation for the WWB variable.

**TABLE 1:** Names, codes, and sources of environmental variables used in the species distribution modeling of *H. verbana*. Uncorrelated variables are highlighted in bold.

Variable category	Environmental variables	Code	Units	Spatial resolution	Source
Climate	**Annual mean temperature**	BIO1	°C		
	Temperature seasonality (standard deviation × 100)	BIO4	SD%		
	**Minimum temperature of the coldest month in the year**	BIO6	°C	2.5 arc min	WorldClim v2.1 https://www.worldclim.org/data/worldclim21.html
	**Annual precipitation**	BIO12	mm		
	Precipitation of the driest month in the year	BIO14	mm		
	**Precipitation seasonality (coefficient of variation)**	BIO15	%		
Topographical	Altitude	ALT	m above sea level	100 m	Tinitaly DEM Sicilia: https://drive.google.com/file/d/10SPlxfjZLG1‐v48p5SYTD1zmCDd4AFp6/view?usp=sharing
Ecological	**The Normalized Difference Vegetation Index (2020‐present)**	NDVI	Fraction	300 m	https://land.copernicus.eu/global/products/ndvi
	**Terrestrial human footprint**	THF	Dimensionless	100 m	https://datadryad.org/dataset/doi:10.5061/dryad.ttdz08m1f
	**Waterways and water bodies**	WWB	Dimensionless	Shapefiles in polygons and lines geometries	https://download.geofabrik.de/index.html

### 2.4 Modeling Techniques and Ensemble Forecasting

The potential distribution of *H. verbana* in Sicily was estimated through the application of multiple statistical and machine learning algorithms, implemented within the biomod2 R package (Thuiller 2003). To optimize model calibration, a total of 60 pseudo‐absence points per dataset were randomly generated, ensuring a balanced ratio between presence and absence records (Barbet‐Massin et al. 2012). These points were subsequently filtered to avoid spatial clustering, allowing only one data point (presence or absence) per grid cell, thereby reducing spatial autocorrelation. Specifically, artificial neural networks (ANN), flexible discriminant analysis (FDA), maximum entropy (MAXENT), and maximum net (MAXNET) were applied. These modeling approaches are well‐established in ecological modeling for their capacity to manage complex, nonlinear relationships and to integrate a variety of environmental predictors (Merow et al. 2013; Thuiller et al. 2019). In particular, machine learning algorithms like ANN and MAXENT, are useful at identifying intricate relationships between species occurrences and environmental factors, often outperforming simpler models when the data exhibit complex, nonlinear patterns. Statistical models such as FDA are valuable for their flexibility and interpretability, especially when incorporating both categorical and continuous variables (Hao et al. 2020).

An ensemble forecasting approach was implemented to integrate the strengths of individual models while minimizing their respective biases. This method allows the combination of outputs from different algorithms, balancing models that generalize well with those that excel at detecting intricate data patterns (Araújo and New 2007). Machine learning models are often recognized for their ability to detect fine‐scale ecological interactions, whereas statistical models are valued for their interpretability and parsimony (Marmion et al. 2009; Hao et al. 2020). A three‐fold cross‐validation procedure was implemented to enhance model reliability and reduce the risk of overfitting. In total, approximately 120 models were generated, accounting for 10 pseudo‐absence datasets, 4 modeling algorithms, and 3 replicate runs. Each model was trained using 80% of the species occurrence data, while the remaining 20% was reserved for model evaluation. The default parameter settings were applied to all models, with the “bigboss” tuning option from the *biomod2* package being used, as recommended by Guéguen et al. (2025). Model performance was assessed using two key metrics, the area under the curve (AUC) of the receiver operating characteristic (ROC) curve and the true skill statistic (TSS). Models achieving AUC values ≥ 0.7 and TSS ≥ 0.4 were considered acceptable in predictive performance and were retained for ensemble modeling (Engler et al. 2011). To account for uncertainty inherent in individual models, consensus ensemble models were constructed by incorporating only those fulfilling the aforementioned performance thresholds (Marmion et al. 2009). Following model calibration, variable importance scores, response curves, and habitat suitability maps were extracted and analyzed to support ecological interpretations. The resulting high‐suitability maps were subsequently used as spatial masks to intersect with the Natura 2000 protected areas layer (https://www.sitr.regione.sicilia.it/download/tematismi/rete‐natura‐2000/). In this case, only highly suitable habitats for *H. verbana* were considered, corresponding to predicted occurrence probabilities ranging from 0.6 to 1.0. This approach allowed calculation of the proportion of overlap between areas of high habitat suitability and existing conservation areas.

All analyses were conducted in accordance with the ODMAP protocol (Zurell et al. 2020), which offers a standardized framework for documenting and reporting species distribution modeling (SDM) workflows. This protocol ensured methodological transparency, reproducibility, and consistency across all phases of the analysis, including data preparation, variable selection, model calibration, and performance evaluation. All analyses were performed in the R software 4.4.3 available at CRAN (http://cran.r‐project.org/), using dismo, biomod2, terra, doParallel, and sf, and QGIS 3.40 software was used for geospatial representation (QGIS Development Team 2024).

## 3 Results

**FIGURE 1:**
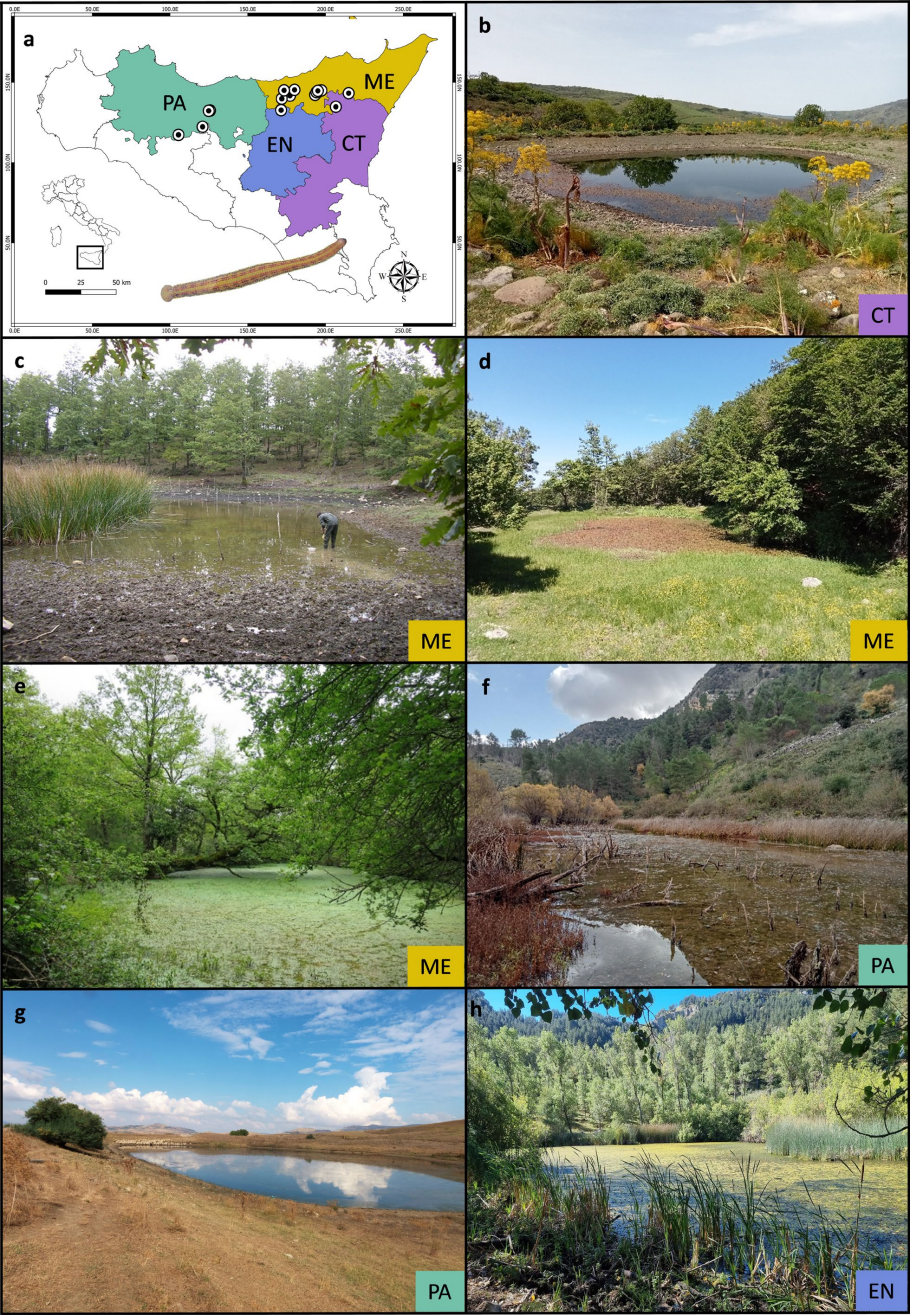
Examples of habitat types where the occurrence of *Hirudo verbana* was confirmed in the frame of our field surveys (see Supporting Information for further details). (a) Map showing the Sicilian provinces where occurrence records (black dots) were collected: Palermo (PA), Enna (EN), Catania (CT), and Messina (ME). (b) Stagno di C.da Bazitta—April 26, 2024 (CT), (c) Stagno di Serra della Testa—September 27, 2008 (ME), (d) Stagno lungo la Dorsale dei Nebrodi—May 24, 2024 (ME), (e) Stagno di Pizzo Luminaria—May 15, 2019 (ME), (f) Gorgo di Sant'Andrea—December 1, 2022 (PA), (g) Stagno di c.da Fontana Murata—September 8, 2023 (PA), and (h) Laghetto Campanito 1—October 2, 2022 (EN, *Source:* https://it.wikiloc.com/percorsi‐escursionismo/riserva‐naturale‐monti‐sambughetti‐e‐campanito‐anello‐115231452 ).

Overall, nineteen verified *Hirudo verbana* occurrence records were included in the analyses (Figure 1 and Table S1). Some old reports lacking accurate locality data as “Gorgo di Fusa,” “Siracusa,” and “Catania” were excluded from the dataset (see Marrone and Canale 2019). Conversely, some previously unpublished records, such as those from “Stagno lungo la Dorsale dei Nebrodi” (Messina) and “Stagno di c.da Fontana Murata” (Palermo), were confirmed as reliable and were thus included in the analyses.

**FIGURE 2:**
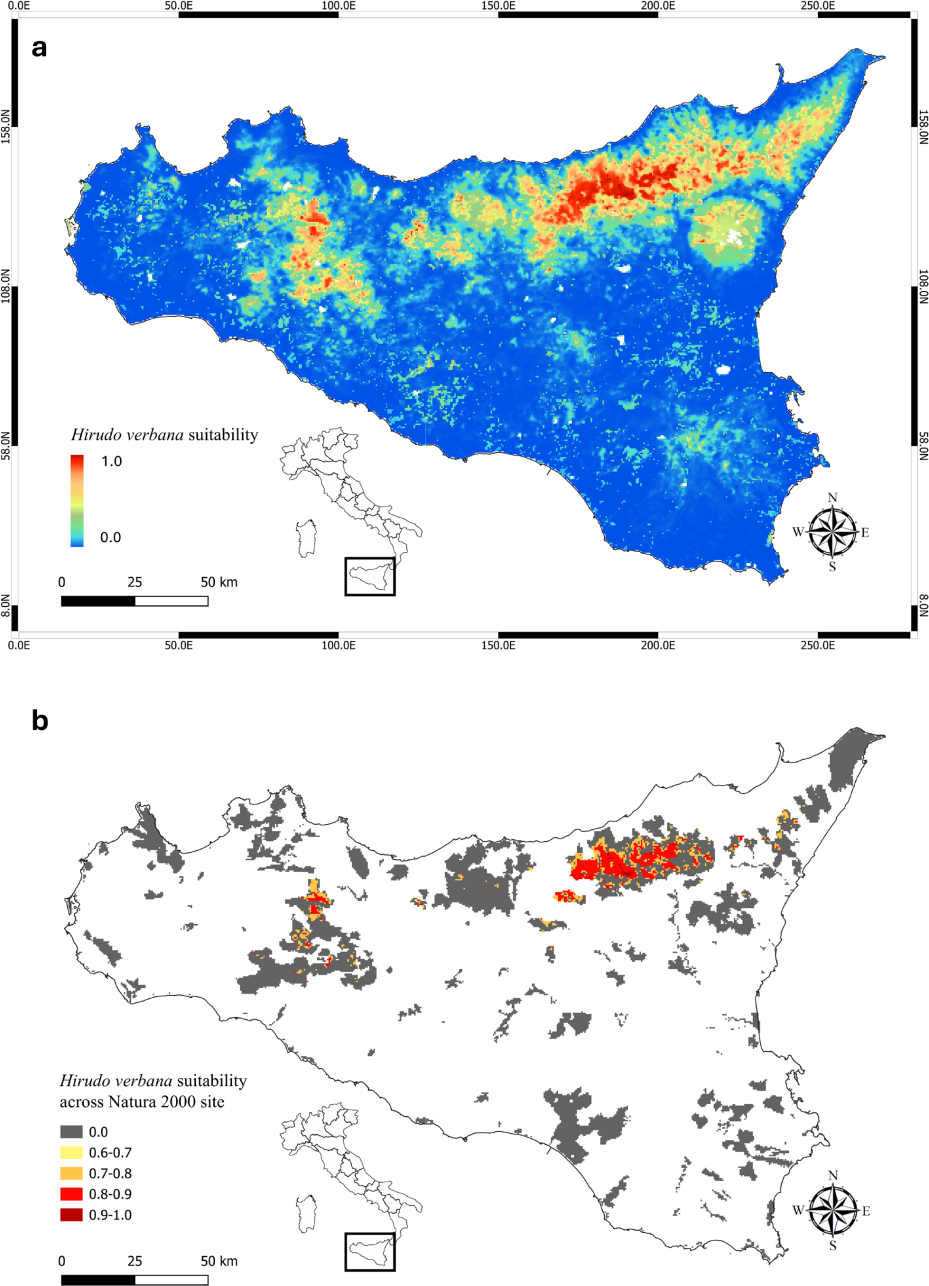
Habitat suitability maps for *Hirudo verbana* across Sicily, showing variations in potential suitable areas within the region (a). Predicted suitability of *H. verbana* within Natura 2000 protected areas in Sicily. The map shows the highest suitability classes, represented as a probability gradient from yellow (0.6–0.7) to dark red (0.9–1.0), while gray areas indicate unsuitability (value = 0.0). Overall, ~35% of the total terrestrial Natura 2000 surface area in Sicily was identified as suitable habitat for *H. verbana* (b).

The final ensemble models showed strong predictive performance, yielding a mean AUC of 0.97 and a TSS of 0.91. These values indicate a high level of model reliability in estimating the distribution of *H. verbana* across Sicily. The potential distribution map of the ensemble model for the Mediterranean medicinal leech on the island, presented in Figure 2, highlights that the species is predominantly associated with upper hill and montane zones. These areas typically support lentic freshwater habitats, such as ponds and marshes, characterized by stable hydrological conditions and dense vegetation, which are essential for the persistence of *H. verbana* populations. Moreover, the final ensemble models identified a suitable habitat for *H. verbana* within Natura 2000 protected areas in Sicily. Analysis of the high‐suitability habitat map (probability of occurrence 0.6–1.0) revealed that ~35% of the terrestrial surface encompassed by Natura 2000 sites overlapped with areas of high suitability for *H. verbana* (Figure 2b). The distribution within this high‐suitability range showed the following pattern: areas with probability 0.8–0.9 covered ~18% of the total surface, representing the most extensive class, followed by zones with probability 0.7–0.8 (~10%), areas with probability 0.9–1.0 (~5%), and marginal patches with probability 0.6–0.7 (~2%). High‐suitability areas were predominantly concentrated in the northern and eastern sectors of the island (Figure 2b).

**FIGURE 3:**
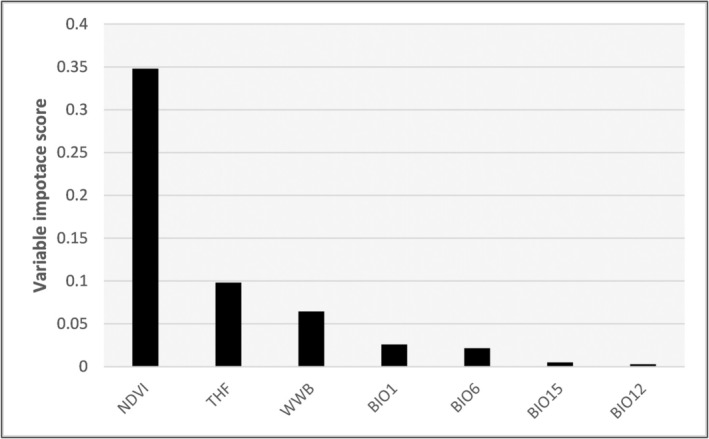
Variable importance measures as defined by Biomod2 for ensemble models. Normalized difference vegetation index (NDVI), terrestrial human footprint (THF), waterways and water bodies (WWB) have the highest importance scores compared with all predictors used in the models. For variable codes and names, see Table 1.

The variable importance analysis revealed that NDVI, THF, and WWB were the three most influential predictors in the model (Figure 3). These variables exhibited significantly higher importance scores compared to the others, highlighting their crucial role in determining the distribution of *H. verbana*.

**FIGURE 4:**
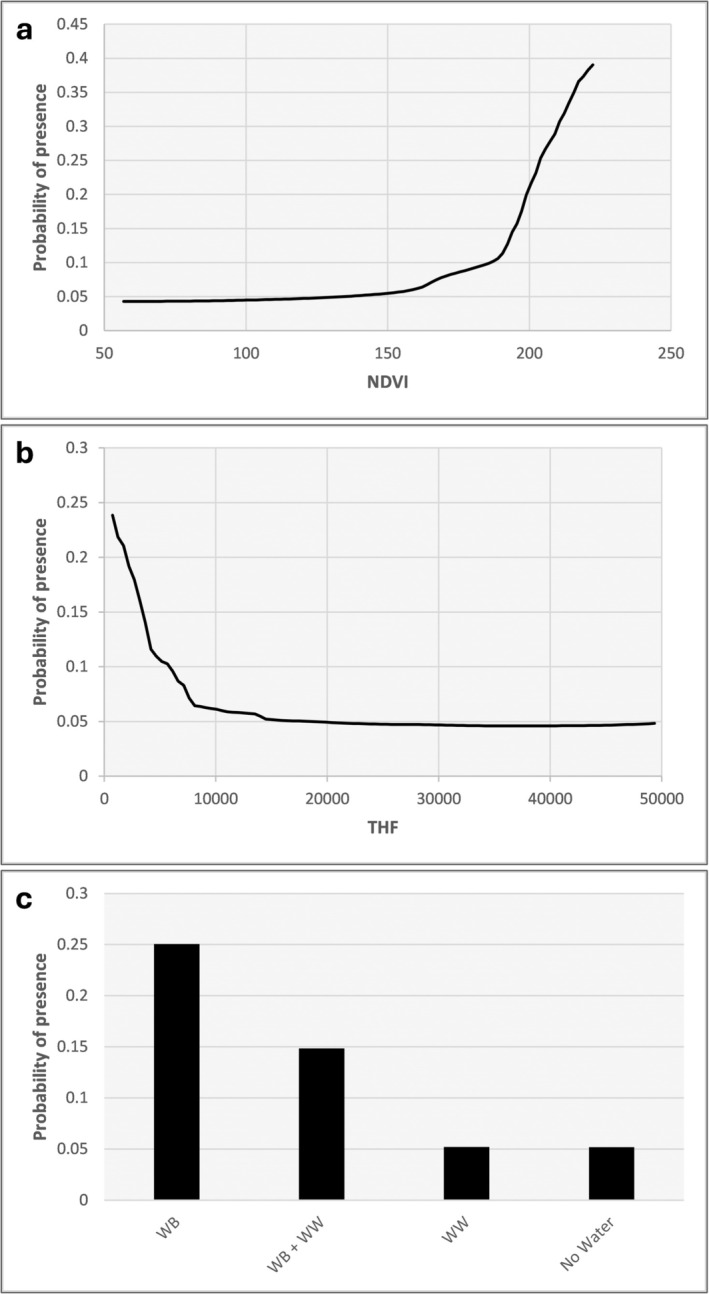
Response curves illustrating the relationship between predictor variables and the relative likelihood of species presence, scaled from 0 to 1. In (a), the probability of presence increases with NDVI (normalized difference vegetation index), particularly after NDVI values exceed 180. In (b), the probability of presence declines with increasing THF (terrestrial human footprint), with the steepest decrease occurring within the first 10,000 units. In (c), the probability of presence varies across water‐related habitat categories, being highest in areas with water bodies (WB), followed by areas with both water bodies and waterways (WB + WW), and lowest in areas with only waterways (WW) or no water.

The probability of species presence increased with higher NDVI, with a pronounced rise observed beyond NDVI values of approximately 180, indicating a strong preference for areas with high vegetation cover (Figure 4a). In contrast, presence probability declined steeply with increasing THF, particularly within the first 10,000 units, suggesting a marked sensitivity to anthropogenic disturbance (Figure 4b). Presence also varied significantly across WWB habitat types. The highest probabilities were associated with areas containing WB, followed by areas with both WB and WW (WB + WW) (Figure 4c). The lowest probabilities were observed in areas characterized by WW or lacking water habitat altogether (No Water), indicating a strong positive association with lentic freshwater environments (Figure 4c). The remaining environmental variables (BIO1, BIO6, BIO15, BIO12) also contributed to the model, but to a lesser extent (Figure 3). Specifically, a strong negative relationship was observed with Annual Mean Temperature (BIO1), where presence probability declined progressively from approximately 0.22 to near 0.05 as mean temperatures increased from around 8°C to 14°C, and remained consistently low at higher values (Figure S2a). Similarly, a decline in presence probability was evident with increasing values of Minimum Temperature of Coldest Month (BIO6) (Figure S2b).

## 4 Discussion

Present results provide useful insights into the distribution and habitat preferences of *Hirudo verbana* in Sicily. Current knowledge regarding the distribution of *Hirudo* species across the national territory of Italy remains fragmented and incomplete, largely due to earlier taxonomic ambiguities and an insufficient sampling effort aimed at censing Hirudinea in the country (Trontelj and Utevsky 2012; Marrone et al. 2021). By focusing on *H. verbana* in Sicily, this study offers a detailed assessment of the ecological preferences of the Sicilian populations of the species, and delineates areas of potential occurrence, thereby enabling the implementation of targeted sampling surveys, which have so far been lacking (Marrone et al. 2021).

The predicted distribution of *H. verbana* across Sicily reveals a clear pattern of habitat suitability predominantly associated with upper hill and montane zones, contrasting with the lowland preferences reported from central and eastern Europe. In Romania and Bulgaria, the species occurs at elevations below 268 m a.s.l. (Gagiu 2010; Todorov et al. 2016; Popa et al. 2024), whereas at lower latitudes, such as southern Turkey and Sicily, populations appear to occupy higher elevation habitats, possibly as a response to increased thermal stress (Sağlam et al. 2016). Exceptions to this pattern are evident in northern Spain, where a lineage reported as “*H. verbana* cf. *verbana*” occurs across a remarkably broad elevational range (4–1517 m a.s.l.), while the allegedly endemic subspecies *H. verbana bilineata* is confined to upper hill and montane habitats (Arias et al. 2021). Such contrasting elevational preferences within the same geographic region suggest the presence of distinct evolutionary lineages with different ecological requirements. Indeed, the record of different *H. verbana* subspecies in Spain indicates that what is currently treated as *H. verbana* likely encompasses multiple lineages with potentially distinct ecological niches. The distributional pattern revealed by our modeling therefore provides some insights into the species' habitat requirements in Sicily, while stressing the importance of integrating fine‐scale phylogeographic analyses with ecological modeling to disentangle the evolutionary and ecological processes shaping distributional patterns in this taxonomically complex group.

Our results also stressed that *H. verbana* habitat suitability in Sicily depends not solely on altitude but on the availability of freshwater environments with stable hydrology, dense vegetation, and low anthropogenic disturbance. The final ensemble model highlighted the significant role of NDVI, THF, WWB in determining the suitable habitat for *H. verbana*. The importance of these variables aligns with previous research on *Hirudo* spp. (Utevsky et al. 2010; Glombová and Schenková 2015), confirming their status as key determinants of habitat suitability. The probability of *H. verbana* presence increases sharply with higher NDVI values, particularly above ~180, reaching a peak around 0.4. This trend indicates a clear preference for areas characterized by dense and well‐structured vegetation. NDVI is often correlated with riparian or aquatic vegetation, which plays a key role in providing microhabitats, shelter, and potential prey for Hirudinea and other animals (Rana et al. 2022). The positive response suggests that *H. verbana* is more likely to occur in ecologically intact freshwater environments where vegetation buffers water temperature, reduces desiccation risk, and supports a more complex food web. Although *H. verbana* has been reported feeding on mammals (Stschegolev 1938, sub “ *Hirudo medicinalis* ”), these hosts are mainly used as dispersal vectors, while amphibians are generally regarded as the primary source of blood meals, as confirmed by several studies (Utevsky et al. 2010; Vecchioni et al. 2025). Consistently, in our Sicilian study sites, as well as in other southern Italian regions (e.g., Abruzzo and Apulia), no feeding on mammals was observed, whereas parasitism on amphibians such as *Hyla intermedia* Boulenger, 1882, *Lissotriton vulgaris meridionalis* (Boulenger, 1882), and *Triturus carnifex* Laurenti, 1768 (Marrone et al. 2021), and occasionally on fish (Vecchioni et al. 2025), was recorded. These prey taxa are strongly associated with aquatic and riparian vegetation (Cinquegranelli et al. 2015; Vecchioni et al. 2025), highlighting that *H. verbana* exploits a range of hosts that share habitats with dense vegetation. This habitat preference might also be affected by physiological constraints, as semiaquatic leeches could show some sensitivity to oxygen availability. Low oxygen levels, such as those occurring in small ponds without aquatic vegetation, may reduce habitat suitability and limit persistence, although current evidence is limited and requires further study (cf. Gagiu 2010). Such dietary preferences and habitat selectivity suggest that the species' distribution might be linked to the structural complexity and ecological integrity of freshwater ecosystems.

The species' probability of presence declines steeply with increasing THF values, suggesting strong sensitivity to anthropogenic disturbance. The curve shows a high probability (~0.24) at very low THF values, dropping rapidly below 0.1 as THF increases beyond ~5000, and approaching near‐zero levels at higher human impact thresholds. This negative relationship underscores the species' reliance on relatively undisturbed freshwater systems and its vulnerability to urbanization, agricultural expansion, pollution, and habitat fragmentation. Nonetheless, Sicilian sites where *H. verbana* is found often have a significant presence of livestock, including cows, sheep, and horses (Marrone and Canale 2019). This suggests that traditional herding practices may support the habitat requirements of these leeches by maintaining the availability of mammalian hosts, which provide high‐energy blood essential for optimal growth and reproductive success (Davies and McLoughlin 1996), and thus should not be considered a form of anthropogenic disturbance for the species. To date, direct evidence of the impact of medicinal leech harvesting on Sicilian populations is lacking. Nevertheless, European trends indicate that overexploitation, wetland drainage, pollution, and land‐use change have historically reduced medicinal leech populations (Elliott and Kutschera 2011; Kutschera and Elliot 2014; Marrone et al. 2024). Sicily experienced extensive reclamation and hydrological‐agricultural transformations from the late XIX century onward (Marrone and NaselliFlores 2022), and the long hiatus in observations during the XX century, followed by sporadic modern detections, is consistent with its historical rarefaction (Utevsky et al. 2010; Sorgi et al. 2011). A comparable case in Sardinia with *H. troctina* illustrates how intensive collection, abandonment of grazing, and wetland loss led to population collapse and extremely scarce XX century records (Whitaker et al. 2004; Marrone et al. 2024, and references therein). While direct evidence for Sicily is unavailable, similar anthropogenic pressures, such as harvesting, habitat modification, and land‐use change, may have likely contributed to the current fragmented and locally rare distribution of *H. verbana*.

*Hirudo* presence also varied significantly across different habitats (WWB). The highest probability of occurrence is associated with areas containing lentic WB (e.g., reservoirs, natural wetlands), followed by mixed zones with both WB and WW (e.g., streams, canals, drains). Suitability declines significantly in areas characterized exclusively by WW and is lowest where no water is present. This pattern confirms the ecological preference of *H*. *verbana* for permanent or semi‐permanent lentic habitats with stable hydrology (Kovalenko et al. 2012; Živić et al. 2015, Popa et al. 2024), although some sites located on the Nebrodi mountains are temporary ponds, possibly representing marginal yet used habitats. These ecosystems provide essential conditions for reproduction, feeding, and movement (Kutschera and Roth 2006; Ceylan et al. 2015; Vecchioni et al. 2025) and a rich amphibian fauna, which is the dominant food source for *Hirudo* where sources of mammalian blood are scarce (Utevsky et al. 2010; Marrone et al. 2021). The relatively lower suitability observed in WW zones may instead be associated with the presence of water current. Except for one site in Tuscany and another in Apulia, where individuals were recorded in small streams, *Hirudo* is typically found in stagnant waters (Marrone et al. 2021). Although the species can occur in low‐flow sections of rivers and canals, such habitats likely fall outside its optimal ecological range.

Finally, despite their relatively low contribution to the overall model performance, some environmental variables still demonstrated a clear response pattern associated with specific climatic predictors. Specifically, a strong negative relationship was observed with BIO1, where the probability of presence declined progressively from approximately 0.22 to near 0.05 as annual mean temperatures increased from around 8°C to 14°C, and remained consistently low at higher values. A similar trend was evident for the minimum temperature of the coldest month in the year (BIO6), with presence probability decreasing steadily from 0.13 at subzero temperatures to 0.04 at values exceeding 8°C. This pattern likely reflects the species' association with upper hill and montane zones in Sicily, where cooler winter temperatures prevail. Although these variables contributed less prominently in terms of model importance, their significant response curves indicate that thermal constraints likely play a role in shaping the species' ecological niche and distribution, particularly in relation to altitudinal compensation within its current latitudinal range. For southern populations (e.g., southern Turkey and Sicily), the occurrence of *H. verbana* at mid to high elevations suggests a certain degree of resilience to climatic variability. However, this pattern may also be influenced by the current distribution of natural areas in Sicily, which are now largely confined to higher elevations. Consequently, the species may not necessarily need high altitudes and low temperatures but rather may be restricted to these areas due to the extensive anthropogenic alteration and destruction of suitable habitats located at lower elevations.

The temperature responses highlighted in this study are broadly consistent with the largely parapatric distribution of *H. verbana* and *H. medicinalis* in Europe, where the two species rarely overlap and only occasionally occur syntopically (Nesemann and Neubert 1999; Utevsky et al. 2010). Such limited coexistence has been interpreted as a sign of ecological segregation, likely influenced by climatic preferences. The negative responses of *H. verbana* to BIO1 and BIO6 in Sicily align with this view. At the same time, the observed altitudinal distribution range of *H. verbana* in southern regions could also result from a combination of other ecological processes, anthropogenic pressures, or intrinsic biological traits such as phenotypic plasticity and genetic factors. These aspects remain currently insufficiently understood but should be considered when interpreting current patterns and anticipating future changes under climate warming.

### 4.1 Conservation Implications for *Hirudo verbana*

The habitat preferences and ecological sensitivity of *Hirudo verbana* underscore its high vulnerability and high conservation value, particularly within Mediterranean landscapes where suitable lentic environments are increasingly fragmented and subject to anthropogenic pressures. The species is strongly associated with areas of elevated ecological integrity, as reflected in habitat suitability models that reveal a spatial overlap with high‐scoring sites in the naturalness evaluation index (NEI) (Baiamonte et al. 2009) and designated conservation areas such as those within the Natura 2000 network (present research). Its marked sensitivity to anthropogenic disturbance, reflected in the decreasing probability of occurrence with higher THF values, combined with its preference for wetlands and well‐vegetated surrounding areas, further highlights its dependence on well‐preserved environments. Additionally, *H. verbana* co‐occurs with diverse aquatic taxa, including plant and animal populations that are widely recognized as indicators of wetland health (Ruffo and Stoch 2006). These traits, together with its documented sensitivity to pollution (Bon et al. 2025), suggest a potential role for *H. verbana* as a bioindicator species. Nonetheless, such an interpretation should be approached with caution, as the species is not consistently present in all high‐quality wetland systems. Rather than serving as a universal bioindicator of wetland ecological integrity, the conservation relevance of *H. verbana* is more clearly reflected in its specific habitat requirements, limited distribution, and inclusion in major legal frameworks such as the Habitats Directive (92/43/EEC) and the Bern Convention. In this light, the species should represent a priority for regional conservation planning, particularly in the context of increasing anthropogenic pressures and habitat fragmentation affecting Mediterranean freshwater ecosystems. Although *H. verbana* has not yet been assessed globally for the IUCN Red List of Threatened Species, national assessments provide useful insights into its conservation status. In Romania, for instance, populations were classified as Vulnerable (VU) (B2b [iii, iv] c [ii]) based on their restricted distribution and ongoing habitat degradation (Popa et al. 2024). A comparable evaluation for Sicily is not yet available; however, our results indicate that local populations are characterized by a fragmented distribution and are exposed to multiple anthropogenic threats, including habitat alteration and loss.

In the absence of direct evidence regarding the impact of harvesting on Sicilian populations, it is plausible that the historical overexploitation that has affected European medicinal leeches may have contributed to their rarefaction on the island. In this context, artificial breeding represents a promising strategy to reduce incentives for wild collection while meeting legitimate demand. In Italy, recent initiatives demonstrate the feasibility of controlled rearing for therapeutic and research purposes, offering a sustainable alternative that can also support awareness‐raising. Coupled with habitat protection and outreach activities, such measures may help promote societal sensitivity toward leech conservation and contribute to the long‐term persistence of *H. verbana* in Mediterranean freshwater ecosystems.

## Author Contributions

**Mirko Liuzzo:** conceptualization (equal), data curation (equal), formal analysis (equal), methodology (equal), software (equal), validation (equal), writing – original draft (equal). **Serge Utevsky:** funding acquisition (equal), supervision (equal), writing – review and editing (equal). **Federico Marrone:** conceptualization (equal), investigation (equal), supervision (equal), visualization (equal), writing – original draft (equal), writing – review and editing (equal).

## Conflicts of Interest

The authors declare no conflicts of interest.

## Supporting information


**Data S1:** ece372410‐sup‐0001‐Supinfo01.docx.

## Data Availability

The datasets generated and analyzed during the current study are available from the sources described in the manuscript and Supporting Information.
