# Supplementary material for: Predictive Distribution Modeling of the Medicinal Leech Hirudo verbana Carena, 1820 (Hirudinea, Hirudinidae) in Sicily: Implications for Conservation
Source: Ecol Evol. 2025 Nov 2;15(11):e72410. doi: 10.1002/ece3.72410 (PMC12580294; doi:10.1002/ece3.72410)
Supplement: Supplementary file 1 — Data S1: ece372410‐sup‐0001‐Supinfo01.docx. [file ECE3-15-e72410-s001.docx]

**FIGURE S1** Elevation map of Sicily depicting the known occurrence localities of *Hirudo verbana* (blue circles) on the island.

**FIGURE S2** Response curves of the ensemble model for predictor variables that contributed less substantially to the species distribution modelling of *Hirudo verbana* in Sicily (a, b, c, d).

**TABLE S1** Occurrence data of Hirudo verbana obtained combining GBIF databases, literature sources, and personal communications.

| **Province** | **Locality** | **Site name** | **Latitude N** | **Longitude E** | **Elevation (m asl)** | **Year** | **N2K Site** | **Source** |
| --- | --- | --- | --- | --- | --- | --- | --- | --- |
| CT | Bronte (CT) | Contrada Bazitta (CT031) | 37.85265 | 14.83128 | 788 | 2010/2024 | ITA070019 | 1, 2 |
| CT | Randazzo (CT) | Stagno di c.da Baiardo (n.a.) | 37.93694 | 14.93388 | 1099 | 2018 | ITA030035 | 3 |
| EN | Nicosia (EN) | Laghetto Campanito 2 (n.a.) | 37.8302 | 14.39109 | 1268 | 2024 | ITA060006 | 4 |
| EN | Nicosia (EN) | Laghetto Campanito 1 (EN035) | 37.83187 | 14.39072 | 1258 | 1985 | ITA060006 | 3 |
| ME | Mistretta (ME) | Urio Quattrocchi (ME022) | 37.901245 | 14.395885 | 1000 | 2019 | ITA030017 | 5 |
| ME | Caronia (ME) | Stagno di Serra della Testa (ME055) | 37.920545 | 14.461728 | 1098 | 2019 | ITA030014 | 5 |
| ME | San Fratello (ME) | Stagno di p.lla Calacudera (n.a.) | 37.926336 | 14.664212 | 1520 | 2011 | ITA030043 | 6 |
| ME | Militello Rosmarino (ME) | Stagno di p.lla Maulazzo (ME036) | 37.935781 | 14.673703 | 1484 | 2021 | ITA030043 | 3 |
| ME | Cesarò (ME) | Stagno Pappanu (ME076) | 37.94158 | 14.68367 | 1559 | 2021 | ITA030043 | 3 |
| ME | Cesarò (ME) | Pozza presso il lago Maulazzo (n.a.) | 37.94318 | 14.67469 | 1430 | 2016 | ITA030043 | 3 |
| ME | Cesaro (ME) | Stagno di Sollazzo Verde (ME027) | 37.95061 | 14.68527 | 1397 | 2016 | ITA030043 | 3 |
| ME | Cesarò (ME) | Stagno lungo la Dorsale dei Nebrodi (ME033) | 37.952271 | 14.699423 | 1340 | 2024 | ITA030043 | 2 |
| ME | Caronia (ME) | Lago Zilio (ME024) | 37.95288 | 14.41408 | 1072 | 2017 | ITA030017 | 3 |
| ME | Cesarò (ME) | Biviere di Cesarò (ME035) | 37.95324 | 14.71469 | 1280 | 1980 | ITA030043 | 3 |
| ME | Caronia (ME) | Stagno di Pizzo Luminaria (ME065) | 37.956226 | 14.498342 | 1104 | 2019 | ITA030014 | 5 |
| PA | Castronovo di Sicilia (PA) | Gorgo di S. Andrea (PA159) | 37.670966 | 13.569222 | 585 | 2019 | ITA020011 | 5 |
| PA | Sclafani Bagni (PA) | Stagno di c.da Fontana Murata (PA188) | 37.722891 | 13.761908 | 551 | 2023 | n.a. | 7 |
| PA | Sclafani Bagni (PA) | Portella di Granza (PA017) | 37.821948 | 13.809168 | 800 | 2019 | n.a. | 5 |
| PA | Sclafani Bagni (PA) | Lago Bomes (PA029) | 37.823823 | 13.820875 | 865 | 2019 | ITA020032 | 5 |
|  |  |  |  |  |  |  |  |  |
| **Sources** |  |  |  |  |  |  |  |  |
| 1 | Utevsky et al. (2010) |  |  |  |  |  |  |  |
| 2 | F. Marrone, unpubl. data |  |  |  |  |  |  |  |
| 3 | Marrone et al., 2021 |  |  |  |  |  |  |  |
| 4 | https://www.inaturalist.org/observations/208560870 | |  |  |  |  |  |  |
| 5 | Marrone & Canale (2019) |  |  |  |  |  |  |  |
| 6 | Sorgi et al. (2021) |  |  |  |  |  |  |  |
| 7 | S. Costa and A. Rallo, unpubl. data |  |  |  |  |  |  |  |
